# A Case of Masked Epilepsy

**Published:** 1886-09

**Authors:** Bonville B. Fox

**Affiliations:** Brislington House


### Clinical Records.

### A CASE OF MASKED EPILEPSY. By Bonville B. Fox, M.A., M.D. Oxon., Brislington House.

The rarity of cases of masked epilepsy invests each one with some clinical importance, while their pathological and medico-legal relationships are of considerable complexity. I therefore venture to hope that the following report may prove of some little interest.

The subject was an unmarried lady, aged 26, who was transferred to Brislington House from another asylum towards the end of last October. Her previous history was bad. In 1869 she had sustained a severe fall from a swing, was much hurt at the time, and ever since suffered from frequent headaches. She had been insane at least twice before, in 1878 and 1880, and recovered on each occasion after about fifteen months' asylum treatment. Of the character of the attacks and nature of the treatment, I have no very accurate account; but she is reported to have been alternately depressed and moody, and excitable. Her family history was poor and suggestive. Her paternal grandmother at some times was "highly nervous," at others "depressed"; two or three paternal uncles were inebriates, one was epileptic. A first cousin had lately destroyed herself; and her mother, the patient's paternal aunt, then became insane. On her mother's side there was comparative freedom from neurotic taint, but strong phthisical predisposition.

The present attack dated from December, 1884, commencing, without adequate exciting cause, in incoherence and excitability. It had continued on and off much the same until she came under our care, ten months subsequently. She brought with her the character of being both dangerous and suicidal, her last medical attendant informing us that she often struck the servants, and that windows appeared to possess an irresistible attraction for her, such numbers had she smashed. On admission, her organs and functions appeared healthy; her fingers were cut, from a final window assault a few minutes before starting on her journey; she seemed free from any delusions, but was moody, taciturn, dull, sluggish, and depressed. She frequently sighed, placed her hand to her head, which was overheated, and complained of pain, weight, and other uneasy sensations therein. The pupils were normal. She was quite docile, fully understood all that was said to her, and associated pleasantly with her fellow-patients. After being in the house three or four days, we saw the first and last specimen of her seizures of violence. Then, without warning or provocation, she dashed her hand through a window, clenched the fragments of glass spasmodically, and, of course, cut herself somewhat severely. Asked for an explanation, all she could say was: "Something comes over me, and I don't know what I am doing." It was particularly noticed, however, that the attack was preceded by pallor of countenance, and was followed by temporary confusion and loss of memory. On considering these symptoms, in association with the family epileptic taint, we thought a close relationship to epilepsy more than probable, and determined to make a systematic trial of treatment on that hypothesis. Accordingly she was promptly placed on scruple doses of bromide of ammonium in sal volatile and camphor-water, thrice daily; her bowels, which were sluggish, were relieved; and she was encouraged to take plenty of regular exercise, occupation, and amusement. This treatment was continued for a month most successfully. She remained dull and lethargic, but there were no further acts of violence. At the end of that time the medicine was stopped; and though for the first few days after its discontinuance there was some increase of cephalalgia, she struggled on without it, maintained her self-control, and a more stable condition of nerve-centres appeared established. Not to be tedious—there was no relapse, but gradual brightening and increase of mental power and activity. After remaining a probationary period of another month or two under our care, she was discharged recovered; better, her father stated, than she had been for the last six years.

Without going into wide and more general questions, I would suggest two points worthy of consideration:—

*(a)* Was this a case of masked epilepsy, cured by anti-epileptic treatment, at all? or, on the contrary, was it merely a case of impulsive insanity, cured by lapse of time and general treatment; and was the marked improvement so soon noticed due to change of scene from one asylum to another, and not to the bromide? I think the point is an open one; but though I express no opinion, on very inadequate data, as to the real nature of the previous attacks of insanity, or indeed as to that of the commencement of the attack of which we saw the end, I am strongly convinced that the aspect of the case, as it reached us, was one of true masked epilepsy. In fact,

I submit that the following symptoms were pretty typical :—

1. Sudden onset of unconsciousness, during which instantaneous, destructive acts were automatically committed : these acts were objectless, uncomplicated and undesigned, nearly always (as we heard) identically the same, and in their nature essentially epileptic.

2. They were preceded and followed by epileptic phenomena; viz., sudden pallor of countenance, loss of memory, and confusion of ideas.

3. They occurred in a neurotic patient, one of a family tainted with epilepsy.

4. Improvement did not follow immediately on admission from change of scene, but was delayed until the treatment by bromide had commenced.

5. The condition of comparative sanity, apart from the paroxysm, favours the theory of epilepsy.

*(b)* Granting, then, that this was a case of genuine masked epilepsy, the question arises: What is the relation of such attacks to the ordinary epileptic discharge ? Do they replace, or do they follow it? Dr. Hughlings Jackson expresses himself as follows in the West Riding Asylum Report for 1875 :—"All kinds of doings after epileptic fits, from slight vagaries to homicidal actions, have one common characteristic—they are automatic; they are done unconsciously, and the patient is irresponsible; hence I use the term mental automatism. . . . I believe there is in such cases, during the paroxysm, an internal discharge, too slight to cause obvious external effects, but strong enough to put out of use, for a time, more or less of highest nervous centres. . . . *The automatism is not, I think, ever epileptic, but always post-epileptic.*" Dr. Jackson's opinion is, of course, entitled to the greatest respect, and it is with the greatest respect that I venture to criticise it. It is obviously impossible to disprove his contention that a discharge too slight for recognition may precede these automatic acts, but the burden of proof lies on him, and I must confess myself at present somewhat sceptical. Such a good authority as Hammond,* while agreeing in the main with Jackson, thinks that such unconscious acts may be substituted for, instead of always following, a seizure. In our patient the attack, no doubt, commenced with pallor of countenance, as most epileptic fits do; but the true discharge, I take it, was synchronous with the violent blow and subsequent spasm, which I presume replaced the tonic and clonic convulsions we more commonly see.

In conclusion, I would remark that this case illustrates three already well-recognised facts :—

1. The extreme importance of procuring a full family history in every case of insanity, as of other disease. Had I not known of the epileptic taint in our patient's relatives, I should have been slower and less certain in my diagnosis of her disease.

2. The advantage of getting a physical basis for the mental symptoms, even where the pathology is still so much a matter of contention as that of epilepsy. How difficult it is to decipher the exact morbid processes that underlie and determine each case of insanity, none can tell but asylum superintendents. The abandonment of empiricism, and the great advance that has been made of late years in the scientific management of insanity, is sufficient proof of the full recognition of the fact that successful treatment must depend upon accurate knowledge.

* *Diseases of the Nervous System*, p. 673.

3. The value of bromides in such cases. This is so well understood that Dr. Savage* assigns as an essential feature to masked epilepsy, that it can be relieved by bromide of potassium. We substituted the ammonium salt, according to our usual practice where bromides have to be given for any considerable time, unless there is sexual excitement, or any advantage to be gained by depressing the patient.

Lastly, the family history exemplifies the kinship of neuroses, and the modification they sometimes undergo in descent. The paternal grandmother was acknowledged to be "highly nervous and depressed." Relatives are very euphemistic in their accounts, and this description might safely be amplified without exaggeration. At best she must have been in a highly neurotic and unstable condition. Of her children, two or three sons were drunkards, one epileptic, and one daughter insane. Her grandchildren included at least one who committed suicide, and our patient.

---

